# A Novel AGV Path Planning Approach for Narrow Channels Based on the Bi-RRT Algorithm with a Failure Rate Threshold

**DOI:** 10.3390/s23177547

**Published:** 2023-08-30

**Authors:** Bin Wu, Wei Zhang, Xiaonan Chi, Di Jiang, Yang Yi, Yi Lu

**Affiliations:** College of Mechanical and Electronic Engineering, Nanjing Forestry University, Nanjing 210037, China; wubin@njfu.edu.cn (B.W.); zhangwnjfu@163.com (W.Z.); chixiaonan0525@163.com (X.C.); jiangdi_jd@163.com (D.J.)

**Keywords:** path planning, narrow channel, Bi-RRT, forklift AGV

## Abstract

The efficiency of the rapidly exploring random tree (RRT) falls short when efficiently guiding targets through constricted-passage environments, presenting issues such as sluggish convergence speed and elevated path costs. To overcome these algorithmic limitations, we propose a narrow-channel path-finding algorithm (named NCB-RRT) based on Bi-RRT with the addition of our proposed research failure rate threshold (RFRT) concept. Firstly, a three-stage search strategy is employed to generate sampling points guided by real-time sampling failure rates. By means of the balance strategy, two randomly growing trees are established to perform searching, which improves the success rate of the algorithm in narrow channel environments, accelerating the convergence speed and reducing the number of iterations required. Secondly, the parent node re-selection and path pruning strategy are integrated. This shortens the path length and greatly reduces the number of redundant nodes and inflection points. Finally, the path is optimized by utilizing segmented quadratic Bezier curves to achieve a smooth trajectory. This research shows that the NCB-RRT algorithm is better able to adapt to the complex narrow channel environment, and the performance is also greatly improved in terms of the path length and the number of inflection points. Compared with the RRT, RRT* and Bi-RRT algorithms, the success rate is increased by 2400%, 1900% and 11.11%, respectively.

## 1. Introduction

With the increasing economic benefits brought to traditional industries by automation and artificial intelligence technology, the need for industrial intelligence transformation is becoming more urgent. Automated Guided Vehicles (AGVs) are increasingly being used in various fields, such as in intelligent warehouses [1,2], automated ports [3,4], unmanned factories [5,6], and in smart agriculture [7,8], to improve the level of intelligence. AGVs have advantages such as their high work efficiency, low cost, and high safety. The research on AGVs includes navigation algorithms [9,10], path planning algorithms [11,12], path tracking algorithms [13,14], and vehicle scheduling algorithms [15,16]. Of these, path planning determines the route by which the AGV will move, and plays a fundamental role in determining the operating costs. Moreover, as the operating environments of AGVs have become increasingly complex, path planning has remained a focus of and challenge in research [11]. Currently, the most commonly used path planning algorithms include the graph-based A* [17] algorithm, the sample-based rapidly exploring random tree (RRT) [18] algorithm, and bio-inspired intelligent algorithms, of which the RRT algorithm is the most widely applied.

The RRT algorithm has several advantages, such as its simple structure, small number of parameters, complete path probability, ability to search effectively in a variety of environments, and the fact that there is no limitation on the spatial dimensions. However, the RRT algorithm also has several drawbacks, including node redundancy, slow convergence speed, and the difficulty of finding the optimal path. The RRT algorithm has been highly anticipated for practical application in path planning in narrow-passage environments, but its current performance does not meet expectations. Many researchers have made improvements to this algorithm, mainly focusing on constraining the sampling space, implementing bi-directional searching, and changing the sampling method. The RRT* [19] algorithm incorporates the concept of reconnecting parent nodes within a circular range and updating the path based on the RRT algorithm. Informed RRT* [20] introduces an elliptical state space based on RRT*, improving the success rate of the target passing through the narrow channel, but with the problem of high time cost. Cyl-HRRT* [21] added cylindrical constraints to the search part of RRT*, thereby reducing the search time, but this method is presented with great challenges in narrow channel environments. An Jaekyu [22] divided the sampling space according to the pipeline, but this was limited to high-dimensional pipeline environments. The Bidirectional RRT (Bi-RRT) [23] algorithm searched the state space by growing two trees simultaneously—from the initial point and from the target point. This was able to help the target pass through the narrow channel, but it was difficult to obtain the optimal path. Klemm added the parent node re-selection and path reconnection concepts of RRT* to Bi-RRT to form the Bi-RRT*algorithm [24]. Although Bi-RRT* reduced the path cost, the time cost was greatly increased. Qureshi [25,26,27] changed the search method of RRT* by integrating the Artificial Potential Field (APF), but this presented problems with local minima. Bi-P-RRT* [28] integrated APF, based on Bi-RRT*. Although the time cost was reduced, there were still many redundant nodes. Fan Jiaming [29] added a target bias strategy to Bi-P-RRT* to further optimize the sampling method. However, the effect of this algorithm when operating in narrow channel environments was not considered. Chai QS [30] improved the success rate of the RRT algorithm by generating a local search tree in a narrow channel, but the path was too long. Ding, J [31] reduced the length of the path by using heuristic search and greedy sampling strategies, but this solution was not optimized for narrow channels. The MIS-RRT algorithm proposed by Huang CM [32] was able to place the robot into the channel by identifying the entrance, but the general adaptability of the algorithm needs to be improved. Li BH [33] combined the Bi-RRT and RRV algorithms in order to be able to adapt to different environments, but there was no reduction in time cost. Numerous scholars have attempted to solve the path planning problem presented by the RRT algorithm in narrow channel environments. However, the effectiveness of these solutions has been limited, and striking a balance between path quality and time cost has proven challenging. In this study, we define the search failure rate threshold (RFRT) concept, thereby establishing a Narrow Channel Bi-directional Rapidly exploring Random Tree (NCB-RRT) algorithm to perform path planning for Automated Guided Vehicles (AGVs). Firstly, the bidirectional RRT algorithm simultaneously creates two search trees, and grows the two trees alternately using a balanced strategy in order to reduce the sampling time. Next, according to the real-time failure rate of sampling, the designed three-stage search strategy, path pruning strategy and Bi-RRT algorithm are fused. The fusion algorithm significantly improves the success rate of operation in narrow channel environments, and reduces the path cost. Then, a piecewise quadratic Bezier curve method is introduced to smooth the path corners. The smoothed path is more in line with the steering motion of AGVs. Finally, compared with the RRT, RRT* and Bi-RRT algorithms, the proposed algorithm can greatly improve the success rate of the AGV passing through narrow channels. Moreover, the path generated by the proposed algorithm has a higher comprehensive performance. The remaining sections of this paper are arranged as follows. The background is discussed in Section 2. In Section 3, we describe the improved algorithm in detail. Section 4 introduces the simulation environment and compares the simulation results. Section 5 presents the conclusions of the paper and illustrates the future research directions.

## 2. Background

### 2.1. Problem Definition

This section uses mathematical symbols to describe the path planning problem of a forklift AGV. The state space is denoted by *S*, and the obstacle space composed of shelves is represented by *S_obs_*. The free search area without obstacles in the space is expressed as *S_search_* = *S*/*S_obs_*. (*S*, *n_start_*, *n_goal_*) defines a path planning problem where the random tree is growing, *n_start_* is the root node of the tree, and *n_goal_* is the target node of the tree. If there is a continuous function *f*: [0, 1] → *S*, and the function is a bounded variation, then the function is a path. If *v* ∈ [0, 1], *f* (*v*) ∈ *S_search_*, then the path *f* is a collision-free path from the root node to the target node.

**Definition** **1** (Feasible path planning)**.** *For the path planning problem (S, n_start_, n_goal_), v is collision-free, v(0) = n_start_, v(1) = n_goal_, and the feasible path is any solution corresponding to v. If no solutions are found, failure is reported.*

**Definition** **2** (Shortest path planning)**.** Given the path planning problem (S, n_start_, n_goal_), after a viable path c(v*) is found, such that c(v*) = min c(v): v is feasible, where c(v) is the full length of the path v. If no solutions are found, failure is reported.

**Definition** **3** (Fast path planning)**.** *Within a certain number of iterations i ∈ R, the optimal solution path with minimum cost is found. Failure is reported if the upper bound for the number of iterations is exceeded.*

### 2.2. Related Work

The rapidly exploring random tree (RRT) [18] algorithm samples the free space in a completely random manner, and gradually establishes the construction of a search tree that extends from the root node to the target node. Different from RRT, the RRT* algorithm adds the parent node re-selection and path update strategies, making the cost of new nodes lower, so as to obtain a path at a lower cost than the RRT algorithm. The Bi-directional Rapidly exploring Random Tree (Bi-RRT) [23] algorithm generates two trees simultaneously, one from the root point and one from the target point. The speed of the bidirectional search is faster, and the success rate of path planning can be effectively improved. The algorithm proposed in this paper is improved based on Bi-RRT, and the pseudo-code for the Bi-RRT algorithm is shown in Algorithm 1.
**Algorithm 1.** Bi-RRT1*T*_1_ ← {*n_start_*}; *T*_2_ ← {*n_goal_*};2*E*_1_ ←∅$;\;E_{2}\;\leftarrow \emptyset$;3**for** each *iter* ∈ [1, *Maxiter*] **do**4 *n_rand_* ← *Sample*(*iter*);5 *n_nearest_* ← *Nearest*(*T*_1_, *n_rand_*);6 *n_new_* ← *Steer*(*n_nearest_*, *n_rand_*);7 **if** *Collision-free*(*n_nearest_*, *n_new_*, *S_obs_*) **then**8  *T*_1_ ←*T*_1_ ∪{*n_new_*};9  *E*_1_ ← *E*_1_ ∪{(*n_nearest_*, *n_new_*)};10  *n′_nearest_* ← *Nearest*(*T*_2_, *n_new_*);11  *n′_new_* ← *Steer*(*n′_nearest_*, *n_new_*);12  **if** *Collision-free*(*n′_nearest_*, *n′_new_*, *S_obs_*) **then**13   *T*_2_ ←*T*_2_ ∪{*n′_new_*};14   *E*_2_ ← *E*_2_ ∪{(*n′_nearest_*, *n′_new_*)};15   *n″_new_* ← *Steer*(*n′_new_*, *n_new_*);16   **if** *Collision-free*(*n′_nearest_*, *n′_new_*, *S_obs_*) **then**17    *T*_2_ ← *T*_2_ ∪{*n″_new_*};18    *E*_2_ ← *E*_2_ ∪{(*n″_new_*, *n′_new_*)};19    *n′_new_* ← *n″_new_*;20   **end if**;21  **if** *n′_new_* = *n_new_* **then Return** *T*_1_, *T*_2_, *E*_1_, *E*_2_;22  **end if**;23 **end if**;24 **if** |*T*_2_| < |*T*_1_| **then**25  *Swap*(*T*_1_, *T*_2_);26  *Swap*(*E*_1_, *E*_2_);27 **end if**;28**end for**29**Return** *T*_1_, *T*_2_, *E*_1_, *E*_2_;

The functions of the pseudo-code are explained as follows:

- *Sample*: Random sample in the environment and return the sample point *n_rand_*.
- *Nearest*(*T*, *n_rand_*): Returns the node closest to *n_rand_* in the tree *T*.
- *Steer*(*n_nearest_*, *n_rand_*): Extend a length from *n_nearest_* to *n_rand_* to get a new node.

In the pseudo-code of the Bi-RRT algorithm, *T*_1_ and *T*_2_ are two trees growing from *n_start_* and *n_goal_*, respectively. *E*_1_ and *E*_2_ record the parent node information of all nodes in *T*_1_ and *T*_2_, respectively. The first tree *T*_1_ is grown by running lines 4–7, and extended to the new node *n_new_*. The expansion approach of the second tree *T*_2_ differs slightly from that of *T*_1_ as it involves two expansion steps. The details of the first step are described in lines 8–12, where the second tree expands in the direction of the new node *n_new_* to obtain the node *n′_new_*. If there is no collision, lines 13–21 are executed to continue expanding in the same direction to obtain *n″_new_* as the second step. Subsequently, the number of nodes in the two trees is compared, and the Swap function is used to exchange the tree with the fewest nodes with the first tree. This process is repeated until the two trees are connected.

## 3. Improved Bi-RRT Algorithm with Search Failure Rate Threshold (SFRT)

**Figure 1:**
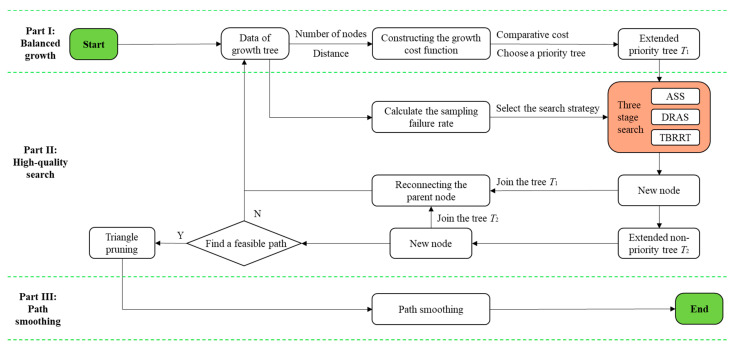
The process of the improved algorithm.

The NCB-RRT algorithm proposed in this paper improves the Bi-RRT algorithm in three main respects. Figure 1 presents a flowchart of the improved algorithm.

- **Balanced growth strategy**: The growth priority of the bidirectional tree of the Bi-RRT algorithm is improved. The number of nodes in each tree and the distance from the target point are used as cost indicators, and the growth of the tree with the lower cost is given priority in order to make the sampling more uniform.
- **High-quality search strategy**: The node closest to the target point on the search tree is selected to carry out the search. Three search strategies were designed to adapt to different environments. The calculated real-time sampling failure rate was compared with the failure rate threshold to determine whether the current environment was simple, complex, or the most complex. When sampling becomes increasingly difficult, the adaptive sector search strategy, the dynamic right angle search strategy, and the target-biased RRT search strategy are gradually implemented to help the algorithm adapt to the obstacle environment. At the same time, the parent node re-selection and the path pruning methods are introduced to reduce redundant nodes and further improve search quality.
- **Path smoothing strategy**: After generating an initial path, the piecewise quadratic Bezier curve method is used to optimize the corners of the path, so that the smoothness of the path is higher and more in line with the steering motion of AGV.

### 3.1. Dual Index-Exchange Search Tree

The RRT algorithm has the problem of requiring a large amount of time when performing AGV path planning. Compared with RRT, the Bi-RRT algorithm has a faster search speed, but there is the risk that the search tree will become trapped by obstacles in narrow channel environments. The unbalanced growth of the search tree is not able to give full play to the advantages of a two-way search. The main improvement presented in this section is that, on the one hand, two trees are extended simultaneously in each iteration; on the other hand, a value function is designed to optimize the birth order of the two trees. The value function is composed of two Indexes: the number of nodes and the distance. The value function of the two trees is calculated according to Equations (1) and (2):

(1) $$Value1=0.5c_{1p}-0.5c_{1l}$$

(2) $$Value2=0.5c_{2p}-0.5c_{2l}$$

where *Value*1 is the growth value of tree *T*_1_, which is extended from the root point, *c*_1*p*_ is the number of nodes of *T*_1_, and *c*_1*l*_ is the nearest distance between *T*_1_ and the end point; *Value*2 is the growth value of tree *T*_2_, which is extended from the end point, *c*_2*p*_ is the number of nodes of *T*_2_, and *c*_2*l*_ is the nearest distance between *T*_2_ and the root point. The value function is recalculated at each iteration. When *Value*1 is greater than *Value*2, *T*_2_ grows preferentially. Otherwise, *T*_1_ grows preferentially.

### 3.2. Search and Optimize Paths

#### 3.2.1. Three-Stage Search Strategy

**Figure 2:**
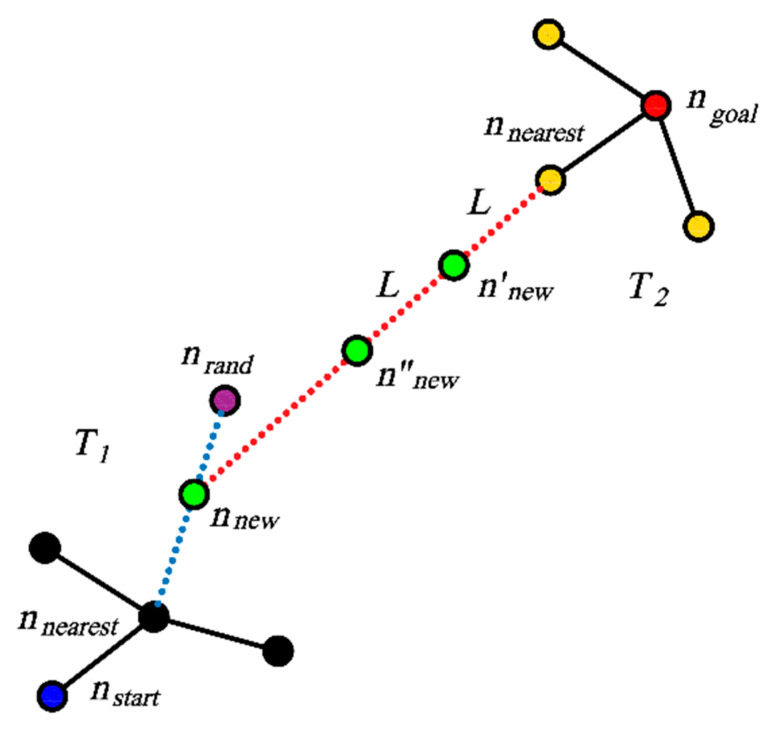
Two-way search tree expansion diagram. *T*_1_ is a random tree that starts from the starting point *n_start_*, *T*_2_ is a random tree that starts from the end point *n_goal_*, *n_new_* is a new node extended by *T*_1_, *n′_new_* and *n″_new_* are nodes grown by *T*_2_.

The steps with which the Bi-RRT algorithm is extended are shown in Figure 2. Three search strategies are designed in this paper based on real-time sampling failure rate. The new search strategy will change the expansion of *n_rand_* and *n_new_* in Bi-RRT, so that the new algorithm can adapt to environments of different complexity. The search strategies are selected according to Equation (3):

(3) $$Sample=\left\{\begin{array}{c} \;adaptive\;sector\;search\;(ASS),\;if\;p\leq p_{1}\\ dynamic\;right\;angle\;search\;(DRAS),\;if\;p_{1}<p<p_{2}\\ \;target\;biased\;RRT\;(TBRRT),\;if\;p\geq p_{2} \end{array}\right.$$

where *p*_1_ and *p*_2_ are the search failure rate threshold, *p*_1_, *p*_2_ ∈ (0, 1), and *p* is the real-time sampling failure rate. The search failure rate threshold (SFRT) is designed as a value that is obtained by dividing the number of failed samples by the total number of samples. The search strategy of each sampling is determined by *p*. When $p\leq p_{1}$, the adaptive sector search (ASS) strategy is selected for the sampling points. When $p_{1}<p<p_{2}$, the dynamic right-angle search (DRAS) strategy is run for sampling; otherwise, the target biased RRT (TBRRT) search strategy is switched to.

**Figure 3:**
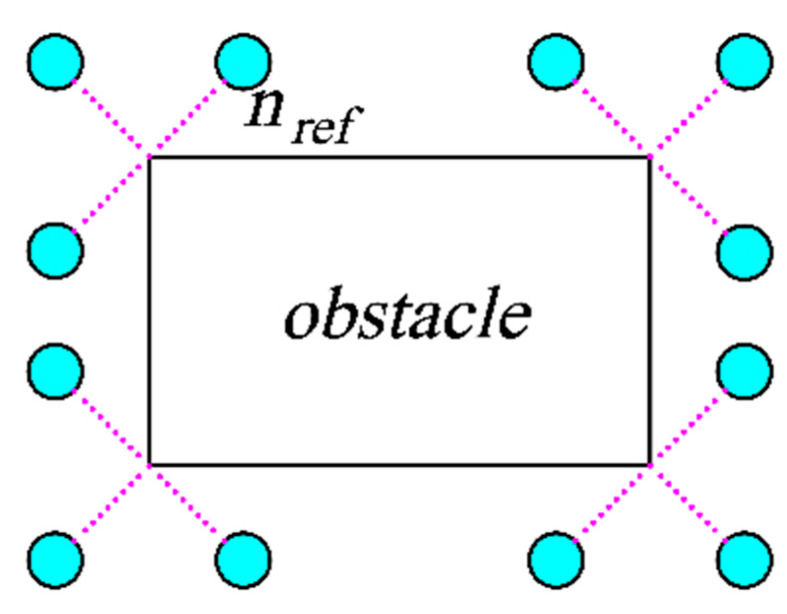
Reference point extension diagram.

The pseudo-code of the ASS strategy is shown in Algorithm 2. Firstly, the algorithm finds the nearest point *n_nearest_* of the search tree to the end point. In line 3, Algorithm 3 returns the set of reference points between *n_nearest_* and *n_goal_*. Then, the reference point in the set that does not collide with *n_nearest_* is denoted as *n_new_* by lines 4–10, and *n_new_* is added to the search tree. If all the points in the set collide, then lines 11–16 are run, and a sampling point is returned from an adaptive sector area defined by Algorithm 4.
**Algorithm 2.** Adaptive sector search strategy1*n_nearest_* ← *n_nearest_*;2*a* ← *Len*(*T*)3*A* ← *Surrounding nodes*(*n_nearest_*, *S*)4**for** each *n_ref_* ∈ *A* **do**5 **if** *Collision-free*(*n_nearest_*, *n_ref_*, *S_obs_*) **then**6  *n_new_* ← *n_ref_*;7  *T* ← *T* ∪{*n_new_*};8  *E* ← *E* ∪{(*n_nearest_*, *n_new_*)};9  **break**
10*b* ← *Len*(*T*)11**if** *b* ! = *a* **then**12 *n_new_* ← *Sample sector*(*n_nearest_*, *n_goal_*, *p*);13 **if** *Collision-free*(*n_nearest_*, *n_new_*, *S_obs_*) **then**14  *T* ← *T* ∪{*n_new_*};15  *E* ← *E* ∪{(*n_nearest_*, *n_new_*)};16 **else then**
17  *n_new_* = *None*;18**Return** *n_nearnest_*, *n_new_*;

**Algorithm 3.** Surrounding nodes1*A* ← ∅; *n_nearest_* ← *n_nearest_*;2*B* ← *Extending nodes*(*S*);3**for** each *n_ref_* ∈ *B* **do**4 **if** *n_ref_* ∈ *S_sur_*(*n_nearest_*, *n_goal_*) **then**5  *A* ←*A* ∪{ *n_ref_* };6 **end if**;7
**end for**
8**Return** *A*;

**Algorithm 4.** Sample sector1*n_nearest_* ← *n_nearest_*; *p* ← *p*;2*a*, *r* ← *Angle*(*n_nearest_*, *n_goal_*, *p*);3*S^’^_sec_* ← *S_sec_*(*a*, *r*);4*n_new_* ← *Sample*(*S^’^_sec_*);5**Return** *n_new_*;

The functions in the pseudo-code are as follows:

- *Len*: Calculate the length of the list.
- *Samplesector*: Sample in a fan-shaped space and return new nodes.
- *Extending nodes*: The collection of a series of reference points, which is composed of reference points (*n_ref_* in Figure 3) that extend around the vertices of each obstacle at a certain length.
- *S_sur_*: A rectangular dynamic search region between *n_nearest_* and *n_goal_*.
- *Angle*: Calculate the central angle and radius of the fan from *n_nearest_* to *n_goal_*.
- *S_sec_*: Plan out the fan-shaped sampling area.

**Figure 4:**
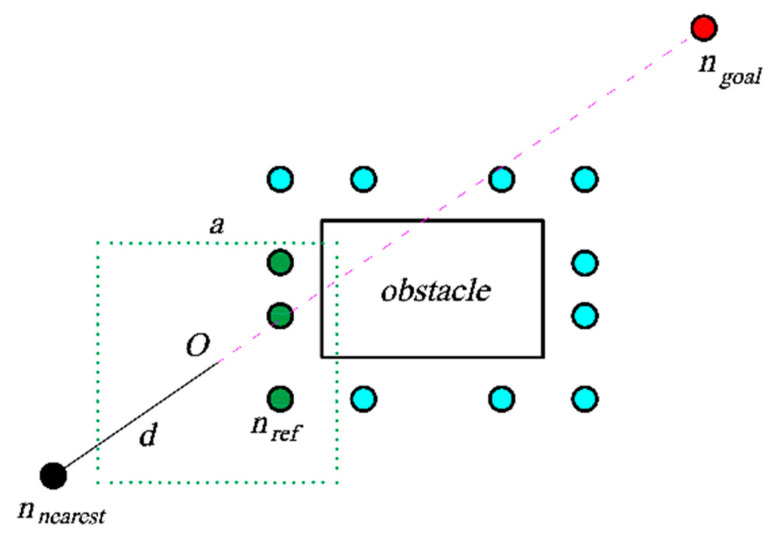
Select the reference points.

**Figure 5:**
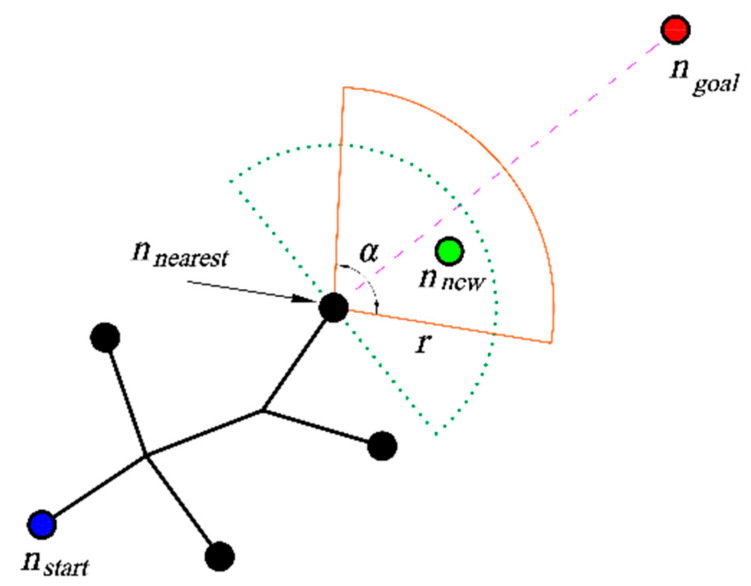
Sector area search diagram.

The generation of reference points is shown in Figure 3. The cyan circle *n_ref_* is the reference point, which is obtained by extending each vertex of the obstacle outward. The dark green points in Figure 4 are the returned reference points, which are distributed in a square with its center between *n_nearest_* and *n_goal_*. *O* is the center of the square, *d* is the distance from *n_nearest_* to *O*, and *a* is the side length of the square area. The dark green circle *n_ref_* is the reference point to return. The fan-shaped sampling area is shown in Figure 5. The line segment composed of the fan-shaped vertex *n_nearest_* and the end point *n_goal_* divides the fan-shaped center angle *α*. The center angle *α* and radius *r* are related to the real-time sampling failure rate *p*. As the failure rate increases, the central angle becomes larger, and the radius becomes smaller. According to Equation (4), *α* is calculated, and *r* is calculated according to Equation (5):

(4) α=π(p+p1)12

(5) r=kL1−p12

where *α* is the central angle, *r* is the radius, *k* is the scaling factor, and *L* is the extended step size of AGV.

**Figure 6:**
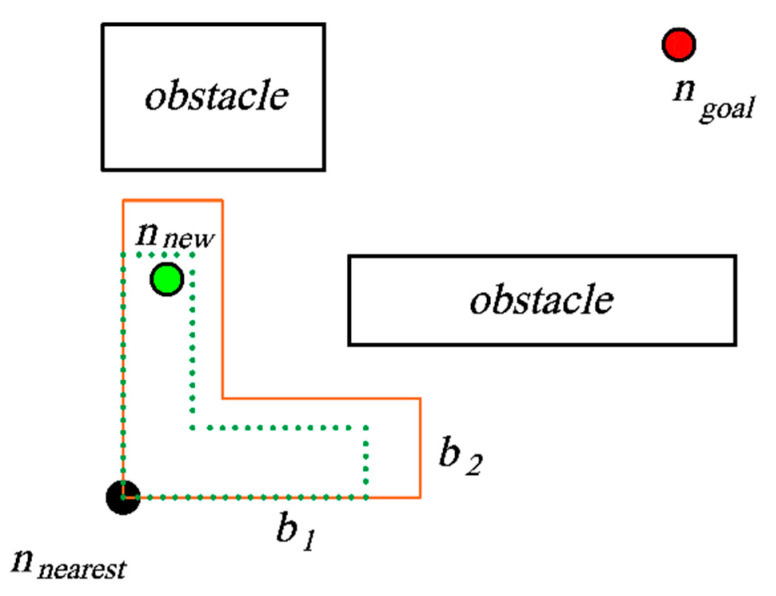
Rectangular area search diagram.

The DRAS strategy and the ASS strategy are basically the same in the algorithm flow. The difference between the two is that the DRAS strategy replaces the sector sampling area in Algorithm 4 with the dynamic right-angle sampling area in Algorithm 5. The right-angle sampling region is expanded towards *n_goal_* with *n_nearest_* as the right-angle vertex. The angle between any right-angle edge and the line segment connected by *n_nearest_* and *n_goal_* is an acute angle. The shape of the right-angled sampling area when the *n_goal_* is at the upper right of the *n_nearest_* is shown in Figure 6. The right-angled area is composed of two identical rectangles that are perpendicular to each other and superimposed. The longer side *b*_1_ and the shorter side *b*_2_ in the rectangle are determined according to Equations (6) and (7):

(6) $$b_{1}=k_{1}L\ln\left(e-p\right)$$

(7) $$b_{2}=k_{2}L\ln\left(e-p\right)$$

where *k*_1_ and *k*_2_ are the gain coefficients, *L* is the extended step size of AGV, *e* is the Euler number, and *p* is the extended failure rate. As *p* keeps increasing, the sample area in Figure 6 gradually shrinks from the shape delineated in orange to that marked in green.
**Algorithm 5.** Sampleright1*n_nearest_* ← *n_nearest_*; *p* ← *p*;2*b*_1_, *b*_2_ ← *rectangle*(*n_nearest_*, *n_goal_*, *p*);3*S^’^_rec_* ← *S_rec_*(*a*, *r*);4*n_new_* ← *Sample*(*S^’^_rec_*);5**Return** *n_new_*;

The functions in the pseudo-code that are different from those in the ASS strategy are described as follows:

- *rectangle*: Calculate the length of two adjacent edges of the rectangle.
- *S_rec_*: Plan out the right-angle sampling area.

The TBRRT search strategy indirectly affects the generation of *n_new_* by adding a target-biased strategy to the RRT to change the selection of *n_rand_*. Random sampling point selection is performed according to Equation (8):

(8) $$n_{rand}=\left\{\begin{array}{cc} n_{goal}, & if\;rand()>m\\ Random, & else \end{array}\right.$$

where *rand*() is a random number between 0 and 1, it is given randomly by the algorithm before TBRRT is run. *m* is the threshold of the target bias. The growth direction of new nodes is determined by *m*. When *rand*() > *m*, the growth tree will grow toward *n_goal_*; otherwise, the growth tree grows randomly in the sampling space.

In this section, we design three search strategies. When the three strategies are used separately, ASS is more advantageous in simple environments. In more complex environments, the success rate of ASS will be reduced by half. DRAS has a high success rate in simple, complex, and more complex environments, and the feasible paths obtained in simple and complex environments are shorter, but DRAS has a longer search time in simple and complex environments than ASS. TBRRT can search in a global environment and find a feasible path without considering the number of iterations, so it can escape from local searches in extreme environments. However, TBRRT requires too many iterations and consumes too much time. Considering the advantage of the fast search time of the ASS in simple environments and the high success rate of DRAS in more complex environments, we use ASS when the SFRT is low, DRAS when the SFRT is high, and TBRRT when the use is higher.

#### 3.2.2. Path Cost Optimization Strategy

The RRT and Bi-RRT algorithms have too many redundant nodes in narrow channel environments, which is an important reason for the high cost of the path. This paper optimizes the problem of node redundancy in the RRT algorithm in two ways. Firstly, with reference to the idea of the RRT* algorithm, the parent nodes re-selection strategy presented in Algorithm 6 is run after sampling new nodes, thus reducing the cost of the new nodes. Secondly, the path is triangularly trimmed after a feasible path has been obtained.

**Figure 7:**
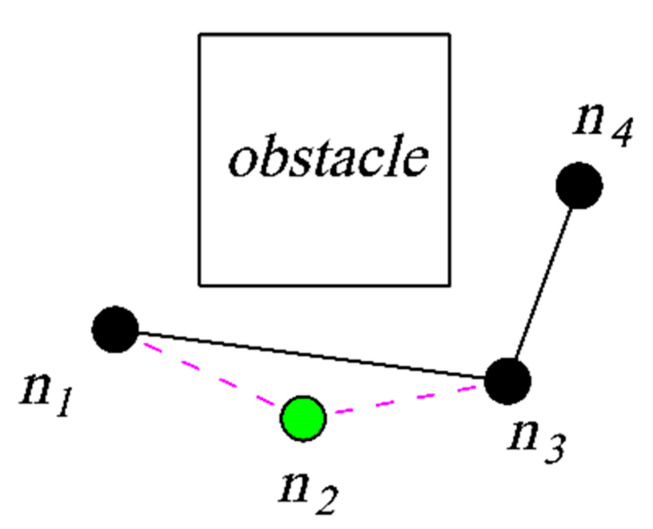
Triangle pruning diagram.

After each new node *n_new_* is obtained, the parent node re-selection strategy presented in Algorithm 6 is run. The strategy defines a neighborhood *S_near_* with *n_new_* as the center of the circle and a fixed length as the radius. Then, the algorithm takes the point *n_min_* in *S_near_* with the smallest path cost after connecting to *n_new_* as the parent node of *n_new_*. The triangle pruning strategy is employed after the algorithm finds a feasible path. At its core is the fact that the sum of the lengths of any two sides of a triangle is greater than the length of the third side. The path pruning process is shown in Figure 7. When the non-collision point n_2_ in the original path is pruned, a new path, n_1_–n_3_–n_4_, is obtained.
**Algorithm 6.** ChooseParent(*S_near_*, *n_new_*, *n_nearest_*)1*n_min_* ← *n_nearest_*;2*Cmindist* ← *C*(*n_near_*) + *Dist*(*n_near_*, *n_new_*);3**for** each *n_near_* ∈ *S_near_* **do**4 *Cdist* ← *C*(*n_near_*) + *Dist*(*n_near_*, *n_new_*);5 **if** *Collision-free*(*n_near_*, *n_new_*, *S_obs_*) **then**6  **if** *Cmindist < Cdist*
**then**7   *Cmindist* = *Cdist*;8   *n_min_* ← *n_near_*;9  **end if**;10 **end if**;11**end for**12**Return** *n_min_*;

### 3.3. Bezier Curve Optimization

Most of the path corners generated by the path planning algorithm are sharp turns, which can negatively impact the operation efficiency and stability of AGVs, and especially of heavy-duty AGVs. Therefore, it is necessary to smooth the corners of the path. Spline interpolation is widely used in path post-processing, but this would greatly change the original search path. At the same time, the Runge–Kutta phenomenon is prone to occur at high orders, and there is a lack of stability in narrow channel environments. Based on the work of Durakli et al. [34], this paper introduces a local path smoothing method based on a quadratic Bezier curve to smooth each path corner. The shape of the quadratic Bezier curve is determined according to Equation (9):

(9) $$B\left(t\right)={\left(1-t\right)}^{2}P_{0}+2t\left(1-t\right)P_{1}+t^{2}P_{2}\;,\;t\in \left[0,1\right]$$

where *P*_0_, *P*_1_, *P*_2_ are control points, *B*(*t*) is a quadratic Bezier curve, and *t* is a time parameter. According to the method designed by Durakli, *P*_0_, *P*_1_ and *P*_2_ are used as control points to generate a continuous arc line. *P*_0_ and *P*_2_ are retained in the new arc line, and *P*_1_ is discarded. By replacing the two straight lines of *P*_0_–*P*_1_ and *P*_1_–*P*_2_ in the original path with new arcs, path smoothing at the inflection point of the path is achieved.

In particular, for the path processed using the ordinary methods, there is a possibility of collision occurring between the path and the obstacle. However, Durakli’s method, described in this section, avoids collisions by adjusting the positions of *P*_0_ and *P*_2_. This makes the work in this section more effective and superior.

## 4. Simulation Results

In this paper, we compared the simulation results of the NCB-RRT algorithm with those of the RRT, RRT* and Bi-RRT algorithms. Kang designed different environments to test the performance of the RRT algorithm [35]. With reference to Kang’s research on simulation environments, we established five simulation environments with which to evaluate the performance of the algorithms.

**Figure 8:**
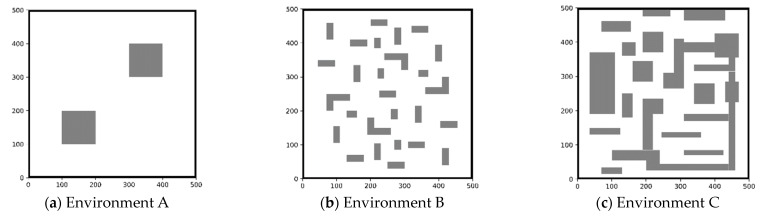

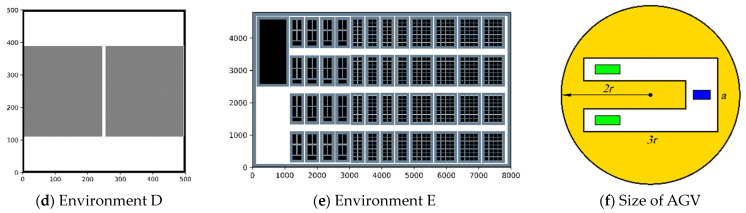
Simulation environment and forklift AGV dimensions. (**a**–**e**) Simulation environments A, B, C, D, and E, respectively. (**f**) Overhead view of the forklift AGV. The vertical length of the AGV is *3r* (*r* = 570 cm), the horizontal length is *a* (*a* = 775 cm), the geometric center of the AGV is the center of the circle. The blue rectangle is the driving wheel that controls the front and back movement of the AGV, and it is able to control the steering movement of the AGV. The green rectangle is the driven wheel.

Firstly, four environments were used to evaluate the performance of the four algorithms. The sizes of the four environments were 500 cm × 500 cm, with the AGV running from (40,40) to (460,460). Figure 8a depicts a simple environment with few obstacles and a large free space. The multi-obstacle environment B is illustrated in Figure 8b, with a more dispersed obstacle distribution to test the convergence speed of the algorithm. Environment C featured concave–convex obstacles, as shown in Figure 8c, posing a significant challenge in obtaining the shortest path. Environment D represented a narrow channel environment, as shown in Figure 8d, where it is difficult of the algorithms to obtain a feasible path.

Next, a more complex environment E was established to test the superiority of the algorithm. This environment was based on an actual map of a railway warehouse, and integrated the characteristics of environments A–D. The size of environment E was 8000 cm × 4800 cm, with the AGV running from (40,40) to (460,460). In Figure 8e, the white part represents the sampling area, and the black rectangles represent the shelves. In order to search for the AGV as a particle, we extend the projection size of the AGV to the shelves. The slate-grey area around the shelves is the extended range, and the obstacles are composed of black and slate-grey rectangles together. The top view of the AGV is shown in Figure 8f, and the slate-grey expansion distance is the radius of the yellow circle.

**Figure 9:**
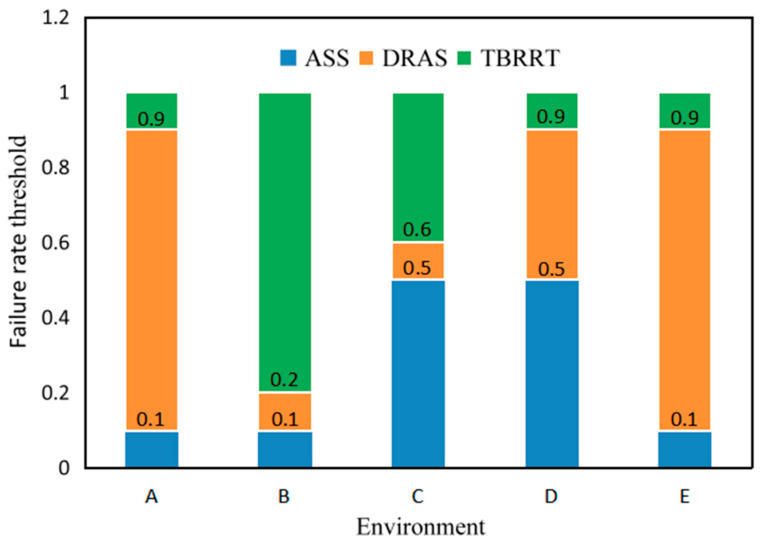
SFRT of the search strategy of NBC-RRT in five environments.

In particular, the search part of the NBC-RRT proposed in this paper consists of three stages. The SFRT is the indicator that divides the three stages. To optimize the performance of the NCB-RRT, different thresholds need to be selected for different environments. Therefore, the combination of the SFRT traverses the distance between 0 and 1 at a step size of 0.1. We designed a cost function to select the group with the lowest average cost in each environment as the final threshold. The cost function is as show in Equation (10):

(10) Cost=0.3node+0.3len+0.2iter+0.2t

where *Cost* is the total cost, node is the average number of nodes, *len* is the average path length, *iter* is the average number of iterations, and *t* is the average running time. Each group was iterated for 20 rounds to obtain the threshold data of Table 1. The role of the three search strategies of NBC-RRT in different environments is shown in Figure 9. A is a simple environment. Although ASS is able to search faster, DRAS accounts for 80% of the whole process because of its greater advantages in terms of path length. The number of obstacles in environment B is large and scattered, causing the algorithm to easily become trapped in local optima; therefore, TBRRT is needed to give full play to its advantages in terms of global search. The barrier-free space of environment C is more continuous than that of environment B, so the path disadvantage of ASS is not obvious compared with DRAS. At the same time, the rapid search of ASS causes the overall advantages of ASS to seem more prominent, such that the use of ASS in environment C reaches 50%. In particular, the bumpy obstacles in Environment C make it easy to get stuck, causing TBRRT to be used for up to 40% of the process. Environment D has two continuous large barrier-free spaces, which makes it easier for ASS to take advantage of its search speed and help the algorithm to reach the narrow channel as soon as possible. The narrow channel is a more complex environment, however, and DRAS can achieve higher success rates in more complex environments under the same number of iterations, so DRAS also plays a great role. In particular, there are few obstacles in environment D, and they are evenly distributed, so the proportion of the process for which TBRRT is used is very small. The barrier-free space of Environment E is small, which makes it impossible for ASS to exert its speed advantage. Similar to environment D, environment E has more home channel space, resulting in DRAS playing a more important role. At the same time, the obstacles in environment E are evenly distributed, so the use of TBRRT is also very low. In addition, it can easily be seen from Figure 9 that the SFRTs of environment A and E are consistent. However, after the above analysis, the reasons for which DRAS has an advantage in these two environments are different. In Environment A, the path advantage of DRAS results in it playing a greater role, while in environment E, it relies more on the ability of DRAS to successfully search in more complex environments.

### 4.1. Performance Testing

**Figure 10:**
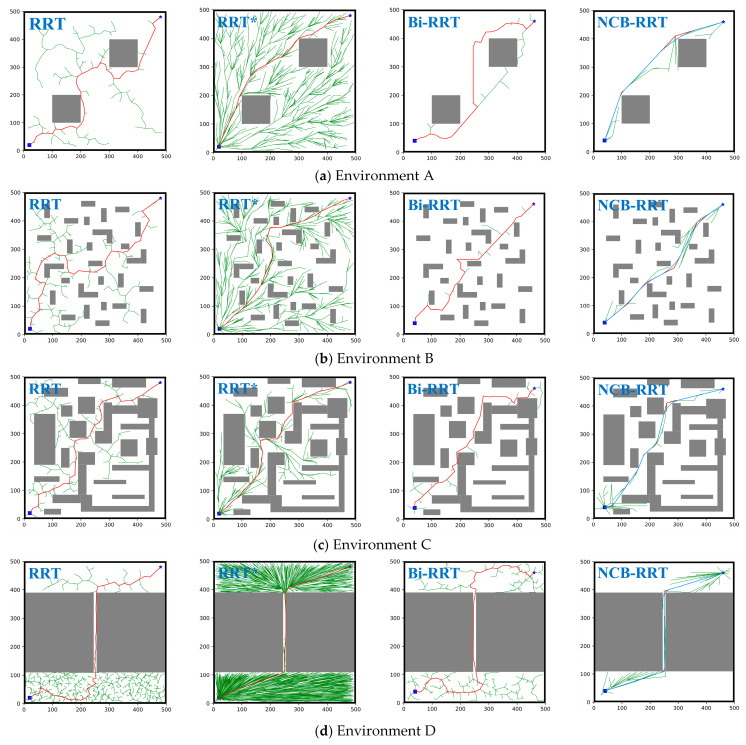
Path results of four algorithms in four environments. (**a**–**d**) Simple environment, multi-obstacle environment, concave–convex environment and narrow channel environment, respectively. In each row, the planning results obtained by the RRT, RRT*, Bi-RRT and NCB-RRT algorithms are presented from left to right, respectively. The blue square is *n_start_*, and the blue pentagram is *n_goal_*.

The paths planned by the four algorithms in the four environments are shown in Figure 10. The root points and the target points are represented by the blue squares and the blue pentagrams, respectively. The growth trees are represented by the green lines, and the obtained paths are indicated by the red lines. The smoothed paths are the blue lines. It can be seen from Figure 10 that the RRT algorithm occupies a large search space. Although RRT was able to obtain a feasible path, that path is long, and there are too many inflection points. Compared with the RRT algorithm, the RRT* algorithm was able to plan a shorter path. However, RRT* needed to be iterated continuously throughout the space, making it difficult to balance the path cost and time consumption. The Bi-RRT algorithm reduced the number of redundant points by using a two-way search, and was able to quickly find a feasible path. However, there are many corners in the obtained path, which may lead to the unstable operation of AGVs in real environments.

In contrast, the red solid line of the fourth image of each row represents the planning results of the NCB-RRT algorithm proposed in this paper, and the blue solid line is the smoothed path. Compared with the RRT, RRT* and Bi-RRT algorithms, the NCB-RRT algorithm was able to effectively deal with a variety of environments with fewer iterations. As shown in Figure 10a, the path obtained using the NCB-RRT algorithm has the fewest path inflection points. It is not difficult to see from Figure 10b,c that the path obtained using the NCB-RRT algorithm has the largest turning angle, causing the AGV to run more smoothly in the multi-obstacle and concave–convex environments. In the narrow channel environment shown in Figure 10d, the NCB-RRT algorithm can still obtain a feasible path quickly. After optimizing the path using the segmented quadratic Bezier curve, the path turning is smoother and more suitable for the AGV’s steering motion.

The average data of the four algorithms after being run 100 times on the simple environment (Figure 10a), the multi-obstacle environment (Figure 10b), the concave–convex environment (Figure 10c), and the narrow channel environment (Figure 10d) are shown in Table 2, Table 3, Table 4 and Table 5, respectively.

From Table 2, Table 3, Table 4 and Table 5, it is evident that as the environment complexity increased, finding a path became more challenging for the RRT and RRT* algorithms. The Bi-RRT algorithm managed to find feasible paths in all four environments, but the path cost was higher than RRT*. The NCB-RRT algorithm proposed in this paper is obviously superior to the other algorithms. Firstly, the NCB-RRT algorithm can still be rapidly implemented in a narrow channel environment due to the balanced growth strategy of the bidirectional tree. Secondly, the three-stage search strategy makes NCB-RRT better able to adapt to different environments, improving the success rate of path finding. Thirdly, NCB-RRT achieves a shorter path cost and fewer turns through the combination of the parent node re-selection strategy and the triangle pruning strategy. The simulation results show that the search ability of NCB-RRT is better than that of other algorithms. It is also notable that NCB-RRT is able to balance performance indicators such as path cost, number of iterations, and number of corners. Of these, corners are defined as places in the path where it is necessary to turn. Compared with RRT, RRT*, and Bi-RRT, NCB-RRT acheives a reduction in path cost of 17.15–40.7%, a decrease in the number of corners by 66.11–94.18%, a decrease in the number of iterations by up to 96.44%, and a reduction in time consumption of up to 92.54%.

### 4.2. Simulation of a Real Warehouse Environment

**Figure 11:**
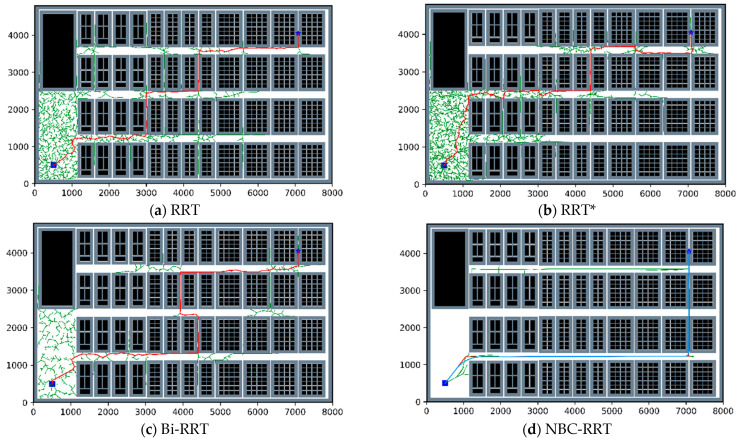
Simulation path diagram in environment E. (**a**–**d**) Simulation results of RRT, RRT*, Bi-RRT and NCB-RRT, respectively. The red line is the searched path, and the blue line is the smoothed path. The blue square is *n_start_*, and the blue pentagram is *n_goal_*.

Environment E is a simulation environment based on an actual warehouse. It consists of a large barrier-free space, a highly uniform distribution of obstacles, and dense narrow channels, making it more complex than environments A–D. As shown in Figure 11, the path planned by the RRT algorithm has a large number of redundant nodes, and the path is not stable. The RRT* algorithm starts to optimize the path after finding a feasible one. The path obtained by RRT* cannot be effectively optimized, because of the large number of iterations consumed during the search. The number of redundant nodes is greatly reduced in the simulated path determined using the Bi-RRT algorithm, but the problems of the instability of the path and the high cost still persist. The planning results of the algorithm proposed in this paper are shown in Figure 11d. The path obtained by the NCB-RRT algorithm has the least redundant points and path turns. After optimizing the path using a Bezier curve, the corner of the path is smoother and more suitable for the steering motion of AGVs.

The average data obtained after 100 runs of the four algorithms are shown in Table 6. The analysis presented in Table 6 shows that the path-finding success rate of the NBC-RRT algorithm proposed in this paper was 11.11–2400% higher than that of the RRT, RRT* and Bi-RRT algorithms, with the average path length being reduced by 13.89–16.83%, the average in running time being decreased by 10.04–81.04%, the average number of iterations being decreased by 18.59–71.85%, and the average number of inflection points being reduced by 93.2–93.58%.

**Figure 12:**
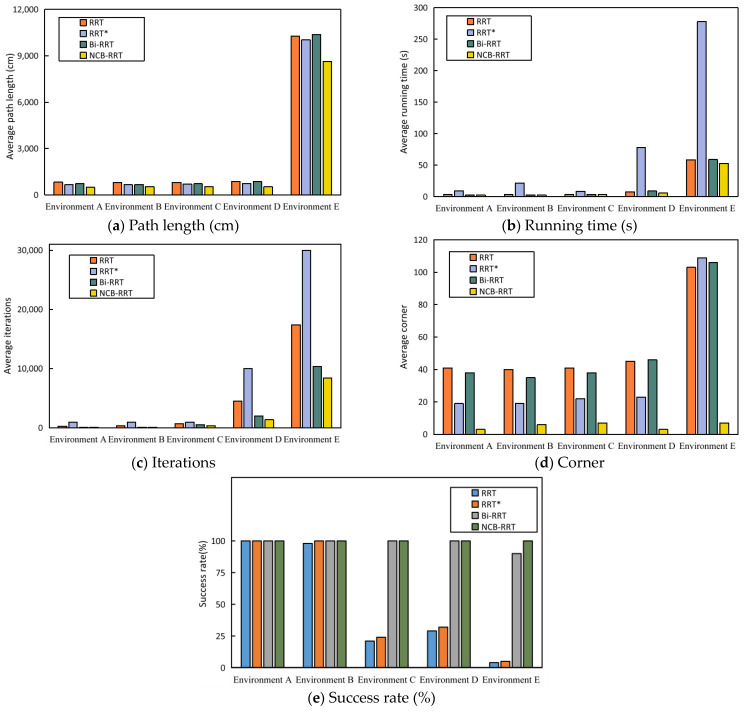
The performance of each algorithm in five environments. (**a**–**d**) Average path length, average time, average number of iterations and average number of corners of RRT, RRT*, Bi-RRT and NCB-RRT, respectively; the lower the value, the better the performance. (**e**) Success rate of path finding; the higher the value, the better the performance.

Figure 12 shows the average performance of the four algorithms in five environments. From Figure 12a–d, it can be seen that, compared with the RRT, RRT* and Bi-RRT algorithms, the proposed algorithm achieved the shortest path and the smallest number of inflection points in a variety of environments. At the same time, the NBC-RRT algorithm is able to balance this performance against time consumption and number of iterations. It is not difficult to see from Figure 12e that with increasingly complex environments, the gap between the success rate of the algorithm proposed in this paper and the success rate of the other algorithms gradually expands. The NCB-RRT algorithm can obtain better paths in five environments, thus demonstrating the superiority of the NCB-RRT algorithm.

### 4.3. Discussion

A superior AGV trajectory will embody characteristics such as minimal path cost, small numbers of corners, minimal time consumption, low number of iterations, and a smooth route. The simulation results show that the balanced strategy helps NCB-RRT solve the problem of tree growth being trapped in narrow channel environments. The proposed balanced growth strategy reduces the consumption and number of iterations of the algorithm. It is not difficult to see from Figure 12b,c that the NCB-RRT algorithm has the lowest time consumption and number of iterations in each environment. The analysis presented in Table 2, Table 3, Table 4, Table 5 and Table 6 shows that compared with Bi-RRT, the time consumption overhead and number of iterations of the NCB-RRT algorithm are reduced by up to 93.4% and 57.93%, respectively.

The three-stage search strategy improves the success rate of NCB-RRT in narrow channel environments, and maintains its search advantage in other environments. The success rate of NCB-RRT is the highest in each environment, as shown in Figure 12e. Compared with the RRT, RRT* and Bi-RRT algorithms, the success rate of the algorithm proposed in this paper represents and increase of up to 2400%, 1900% and 11.11%, respectively. The introduction of the parent node re-selection strategy allows NCB-RRT to initially optimize tree growth during the search process. By integrating the triangle construction strategy, NCB-RRT is able to trim the path well both during and after the search process. The fusion of the two methods solves the problems of there being too many path turns and too much instability in the RRT algorithm. As seen in Figure 10 and Figure 11, the paths obtained using NCB-RRT have the fewest inflection points. The analysis presented in Table 2, Table 3, Table 4, Table 5 and Table 6 shows that the number of corners using the proposed algorithm is much smaller than using the other algorithms. Compared with the RRT, RRT* and Bi-RRT algorithms, the number of corners obtained using the NCB-RRT algorithm represents a reduction by 94.18%, 93.75% and 94.24%, respectively.

By optimizing the corners using a piecewise quadratic Bezier curve to make the path smoother, the difficulty of the path obtained not being suitable for AGV operation is solved. It can be seen from the blue lines in Figure 10 and Figure 11 that the optimized paths allow smoother transit at the corners, thus meeting the expected requirements.

It is not difficult to see from Figure 10 and Figure 11 that the algorithm proposed in this paper is able to find a better path in a narrow channel environment, while still having good performance in other environments. However, compared with Bi-RRT, NCB-RRT adds more strategies, which results in it having some weaknesses. It can be seen from Table 3 that the running time of NCB-RRT is slightly higher than that of Bi-RRT in relatively simple environments.

## 5. Conclusions

In this paper, the NCB-RRT algorithm with sampling SFRT was proposed to solve the path planning problem for AGVs in narrow channels. Firstly, two alternating random trees were established using the balance strategy to search for new nodes. Then, we designed a three-stage search strategy to improve the quality of the sampling points. The new search method demonstrated a higher success rate and a greater adaptability than the RRT, RRT* and Bi-RRT algorithms in an actual warehouse environment with narrow channels. Next, we added the parent node re-selection strategy following the generation of new nodes, and the path pruning strategy was performed after the generation of the initial path. The problems of faced by the RRT and RRT* algorithms of having too many redundant nodes, too many iterations and excessive path length were solved by the integrated path pruning strategy. Finally, the segmented quadratic Bezier curve was used to further optimize the path, so that the path corner was smooth and more in line with the driving requirements of the AGV. The simulation results showed that the proposed algorithm is able to effectively reduce the path cost and improve the search efficiency through complex narrow channel environments. However, this algorithm only takes the path planning of a single AGV into consideration. In actual storage application, increasing numbers of collaborating AGVs are required for transportation, and this will be the next topic to be studied.

## Figures and Tables

**Table 1:** SFRT for NBC-RRT.

Environment	A	B	C	D	E
*p*_1_	0.1	0.1	0.5	0.5	0.1
*p*_2_	0.9	0.2	0.6	0.9	0.9

**Table 2:** Simulation data in environment A.

Algorithm	Success Rate (%)	Average Path Length (cm)	Average Running Time (s)	Average Iterations	Average Corners
RRT	100	830.3207	3.3379	310	41
RRT*	100	659.0076	9.4609	1000	19
Bi-RRT	100	725.4594	2.8425	85	38
NCB-RRT	100	492.3891	2.6257	36	3

**Table 3:** Simulation data in environment B.

Algorithm	Success Rate (%)	Average Path Length (cm)	Average Running Time (s)	Average Iterations	Average Corners
RRT	98	810.1111	3.5699	367	40
RRT*	100	658.5574	21.9250	1000	19
Bi-RRT	100	676.1681	2.9837	51	35
NCB-RRT	100	545.6007	3.0067	52	6

**Table 4:** Simulation data in environment C.

Algorithm	Success Rate (%)	Average Path Length (cm)	Average Running Time (s)	Average Iterations	Average Corners
RRT	21	818.5157	3.6746	723	41
RRT*	24	703.6255	8.7475	1000	22
Bi-RRT	100	722.3770	3.5743	569	38
NCB-RRT	100	547.1600	3.5157	346	7

**Table 5:** Simulation data in environment D.

Algorithm	Success Rate (%)	Average Path Length (cm)	Average Running Time (s)	Average Iterations	Average Corners
RRT	29	885.4526	8.0130	4567	45
RRT*	32	738.1071	78.4928	10,000	23
Bi-RRT	100	862.8697	8.8575	2002	46
NCB-RRT	100	526.0580	5.8575	1389	3

**Table 6:** Simulation data in environment E.

Algorithm	Success Rate (%)	Average Path Length (cm)	Average Running Time (s)	Average Iterations	Average Corner
RRT	4	10,285.2113	58.6121	17,421	103
RRT*	5	10,034.2300	278.0813	30,000	109
Bi-RRT	90	10,388.9534	58.9138	10,374	106
NCB-RRT	100	8640.9002	52.7265	8445	7

## Data Availability

No new data were created or analyzed in this study. Data sharing is not applicable to this article.
